# The Swan-Song of a Great Matron-In-Chief

**Published:** 1919-01-04

**Authors:** Sidney Browne

**Affiliations:** Matron-in-Chief.


### THE SWAN-SONG OF A GREAT MATRON-IN-CHIEF.

The following is a copy of the letter sent by the Matron-in-Chief of the Territorial Force Nursing Service to the members of the Force from the War Office, London, dated Christmas 1918:—

Dear Members of "Ours" and those who serve with us.

This is the happiest Christmas we have had together since the war began. Deep down in our hearts there is great thankfulness that God has given us the victory over our enemies: there is a joy we can hardly express in words. Perhaps it comes partly from the happiness of those brave, valiant soldiers who have given their lives for their country, and who are rejoicing with us that the battle has been won, and that their deaths have not been in vain.

We are all feeling the strain of these long years of war. Some of us are quite tired out and are longing for a rest, but we are very happy in spite of outward circumstances; the Christmas bells are ringing in our hearts, and it is indeed peace on earth, good will to men of good will.

We shall shortly be demobilised and return to our civil occupations, but we shall never forget our active service. It is just as much real active service for doctors and nurses at home as it is abroad, as we are not under training, but we are giving all our strength to fight against disease and death.

It will be a renewed, better world to which we return, we hope. There is to be a "League of Nations" to make peace as permanent as it can be on earth. Nations are formed of individuals, so we can each do our utmost to bring in a new spirit of love and fellowship. We have learned something of the value of this spirit in our Army work which we can never forget. We have been comrades and friends with each other, and with the brave men we have nursed, and with those under whom we have served, and it is a great happiness to have such a bond of union.

You have done your work splendidly in this war, and won the respect and admiration of all with whom you have come into contact. Many members of the T.F.N.S. and V.A.D.s. have given their lives for their country in a spirit of entire self-sacrifice, and you have been wonderfully brave, and have not failed in your duty in times of danger and peril. I am very proud of the records which have been sent to me, but more than all of the knowledge that you think little of what you have done and do not desire any praise or reward; but what you appreciate most is to know that you have helped thousands of our men, whose poor wounded bodies you have tended with such gentleness and skill, and inspired them with fresh courage and hope. Put a high ideal before you, and do your future service in a greater strength than your own, and your life will be for the betterment of the world, and you will know the true happiness of those who try faithfully, in small things as well as in great, to follow and serve the great King. It is His pleasure to call us to serve Him faithfully in this life, so that when the battle of life is over we may have joy and gladness for ever.

This is the last Christmas letter I shall have the pleasure of writing to you, as we sh[illegible] be demobilised after peace is signed, and with all my heart I wish you success and happiness in your future life.

Yours most sincerely,
Sgd. SIDNEY BROWNE,
*Matron-in-Chief.*

